# Supplementary material for: DNA methylation estimation using methylation-sensitive restriction enzyme bisulfite sequencing (MREBS)
Source: PLoS One. 2019 Apr 4;14(4):e0214368. doi: 10.1371/journal.pone.0214368 (PMC6448829; doi:10.1371/journal.pone.0214368)
Supplement: S1 File — (PDF) [file pone.0214368.s001.pdf]

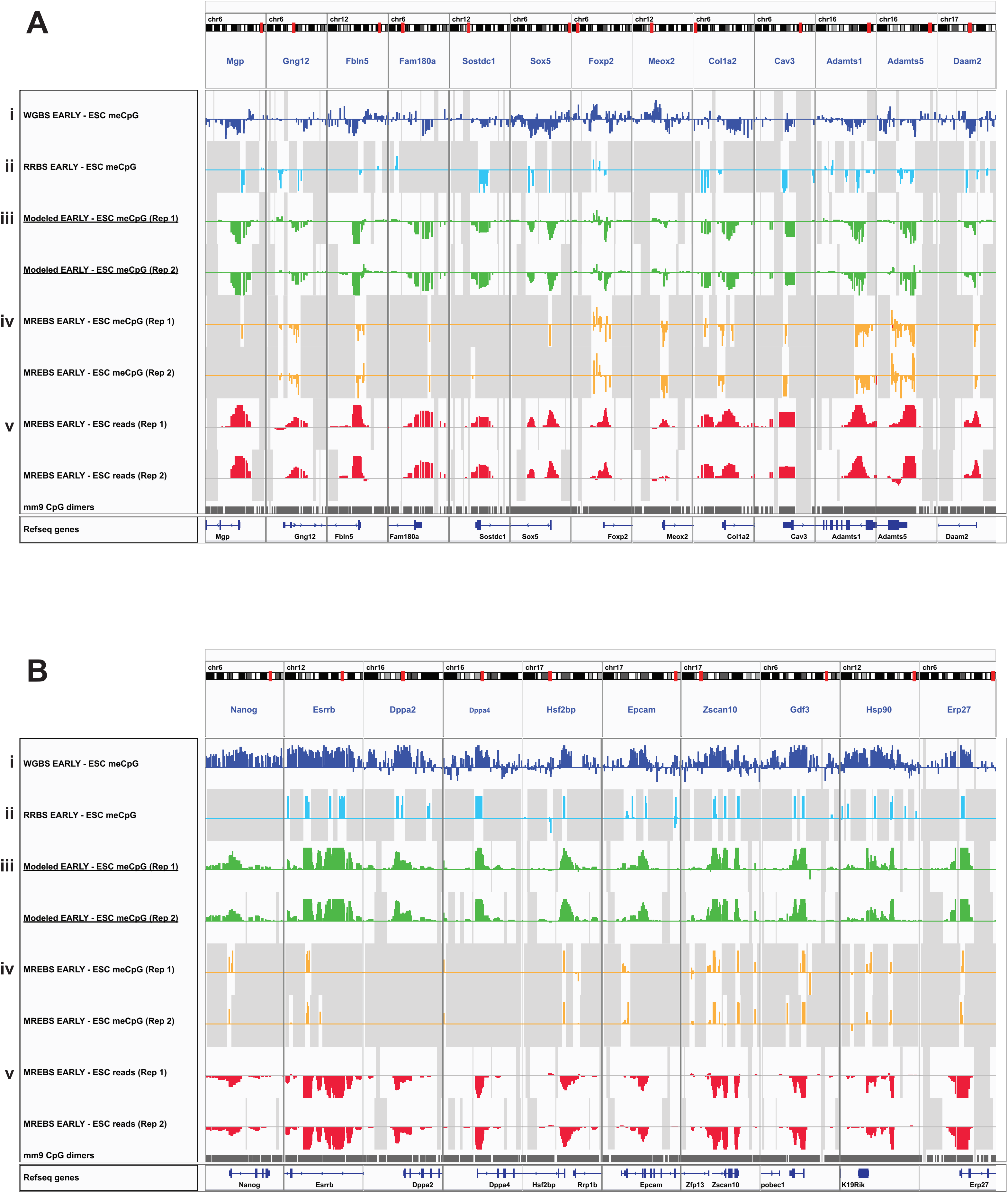
A
chr6
chr12
chr16
chr17
Mgp
Gng12
Fbln5
Fam180a
Sostdc1
Sox5
Foxp2
Meox2
Col1a2
Cav3
Adamts1
Adamts5
Daam2
i WGBS EARLY - ESC meCpG
ii RRBS EARLY - ESC meCpG
iii Modeled EARLY - ESC meCpG (Rep 1)
Modeled EARLY - ESC meCpG (Rep 2)
iv MREBS EARLY - ESC meCpG (Rep 1)
MREBS EARLY - ESC meCpG (Rep 2)
v MREBS EARLY - ESC reads (Rep 1)
MREBS EARLY - ESC reads (Rep 2)
mm9 CpG dimers
Refseq genes
B
Nanog
Esrrb
Dppa2
Dppa4
Hsf2bp
Epcam
Zscan10
Gdf3
Hsp90
Erp27
Rrp1b
Zfp13
pobec1
K19Rik

MRE restriction enzyme	4mer CpG context	Instances in mm9 genome (1 strand)
	ACGT	1,756,359
HpaII*	CCGG	1,594,148
	CCGT	1,454,486
	ACGG	1,449,336
	TCGG	1,404,375
	CCGA	1,401,476
	TCGT	1,392,167
	TCGA	1,391,828
	ACGA	1,391,206
	GCGT	1,258,753
	ACGC	1,255,903
AciI	CCGC	1,251,553
	GCGG	1,250,525
Hin6I	GCGC	1,102,589
	TCGC	995,280
	GCGA	992,773
	NCGA	7
	NCGC	4
	ACGN	3
	CCGN	3
	GCGN	2
	NCGG	2
	NCGT	1
	Total	21,342,779

Supplementary Table A

	Total Mapped Reads	Mean CpG coverage depth
**WGBS EARLY**	429,374,384	7.80
**WGBS ESC**	391,724,853	7.23
**RRBS EARLY**	12,826,209	12.49
**RRBS ESC**	18,131,716	18.88
**MREBS EARLY Rep1**	11,963,716	5.72
**MREBS EARLY Rep2**	12,400,629	5.78
**MREBS ESC Rep1**	11,835,343	6.35
**MREBS ESC Rep2**	12,222,192	6.28

Supplementary Table B

	CpG dimers*	%	
**WGBS EARLY DNAme**	17,202,917	80.6%	CpG dimers with 5X coverage
**WGBS ESC DNAme**	17,022,903	79.8%	
**RRBS EARLY DNAme**	1,289,663	6.0%	
**RRBS ESC DNAme**	1,358,529	6.4%	
**MREBS EARLY Rep1 DNAme**	962,559	4.5%	
**MREBS EARLY Rep2 DNAme**	924,551	4.3%	
**MREBS ESC Rep1 DNAme**	918,508	4.3%	
**MREBS ESC Rep2 DNAme**	973,441	4.6%	
**MREBS EARLY Rep1 Counts>0**	14,695,688	68.9%	Read falling within 1kb around CpG dimer
**MREBS EARLY Rep2 Counts>0**	14,718,855	69.0%	
**MREBS ESC Rep1 Counts>0**	13,602,796	63.7%	
**MREBS ESC Rep2 Counts>0**	13,947,144	65.3%	
**MREBS EARLY Rep1 Counts>=2**	10,250,065	48.0%	
**MREBS EARLY Rep2 Counts>=2**	10,315,026	48.3%	
**MREBS ESC Rep1 Counts>=2**	8,987,596	42.1%	
**MREBS ESC Rep2 Counts>=2**	9,227,587	43.2%	
**MREBS EARLY Rep1 Counts>=5**	5,317,311	24.9%	
**MREBS EARLY Rep2 Counts>=5**	5,387,683	25.2%	
**MREBS ESC Rep1 Counts>=5**	4,696,304	22.0%	
**MREBS ESC Rep2 Counts>=5**	4,659,571	21.8%	
**MREBS EARLY Rep1 Counts>=10**	3,557,311	16.7%	
**MREBS EARLY Rep2 Counts>=10**	3,610,365	16.9%	
**MREBS ESC Rep1 Counts>=10**	3,389,630	15.9%	
**MREBS ESC Rep2 Counts>=10**	3,352,308	15.7%	

Supplementary Table C

	CpG dimers with 5X coverage in both EARLY and ESC	%
**WGBS**	16,113,172	75.5%
**RRBS Rep1**	1,204,249	5.6%
**MREBS Rep1**	649,614	3.0%
**MREBS Rep2**	665,431	3.1%

	CpG dimers with at least 2 reads in the surrounding 1 kB region in at least one sample	%
**MREBS Rep1**	12,542,720	58.8%
**MREBS Rep2**	12,746,080	59.7%

Supplementary Table D

	Model 1	Model 2		Model 3		Model 4	
	RRBS-based differential DNA methylation	MREBS conversion-based differential DNA methylation + differential read counts		MREBS conversion-based differential DNA methylation only		MREBS differential reads counts only	
		Rep1	Rep2	Rep1	Rep2	Rep1	Rep2
**B0 (intecept)**	5.29	5.09	4.92	5.45	5.01	2.61	2.73
**B1**	0.75	0.53	0.54	0.63	0.64	-0.38	-0.38
**B2**	N/A	-0.09	-0.09	N/A	N/A	N/A	N/A

Supplementary Table E
